# Occurrence of the amphibians in the Volga, Don River basins and adjacent territories (Russia): research in 1996-2020

**DOI:** 10.3897/BDJ.8.e61378

**Published:** 2020-12-29

**Authors:** Alexander Ruchin, Oleg Artaev, Elvira Sharapova, Oleg Ermakov, Renat Zamaletdinov, Vjacheslav Korzikov, Ivan Bashinsky, Alexey Pavlov, Anton O Svinin, Alexander Ivanov, Vasily Tabachishin, Anastasiya Klenina, Svetlana Ganshchuk, Nikolai Litvinov, Nikolai Chetanov, Andrei Vlasov, Olga Vlasova

**Affiliations:** 1 Joint Directorate of the Mordovia State Nature Reserve and National Park "Smolny", Saransk, Russia Joint Directorate of the Mordovia State Nature Reserve and National Park "Smolny" Saransk Russia; 2 Papanin Institute for Biology of Inland Waters Russian Academy of Sciences, Borok, Russia Papanin Institute for Biology of Inland Waters Russian Academy of Sciences Borok Russia; 3 Municipal budgetary institution of the additional education "Station of young naturalists" in Sarov, Sarov, Russia Municipal budgetary institution of the additional education "Station of young naturalists" in Sarov Sarov Russia; 4 Penza State University, Penza, Russia Penza State University Penza Russia; 5 Kazan Federal University, Kazan, Russia Kazan Federal University Kazan Russia; 6 Federal Hygienic and Epidemiological Center in Kaluga Region of Rospotrebnadzor, Kaluga, Russia Federal Hygienic and Epidemiological Center in Kaluga Region of Rospotrebnadzor Kaluga Russia; 7 A.N. Severtsov Institute of Ecology and Evolution of RAS, Moscow, Russia A.N. Severtsov Institute of Ecology and Evolution of RAS Moscow Russia; 8 Volzhsko-Kamsky National Nature Biosphere Rezerve, Sadoviy, Russia Volzhsko-Kamsky National Nature Biosphere Rezerve Sadoviy Russia; 9 Mari State University, Yoshkar-Ola, Russia Mari State University Yoshkar-Ola Russia; 10 A.N. Severtsov Institute of Ecology and Evolution of RAS, Saratov, Russia A.N. Severtsov Institute of Ecology and Evolution of RAS Saratov Russia; 11 Samara Federal Research Center of Russian Academy of Sciences, Institute of Ecology of the Volga River basin of RAS, Togliatti, Russia Samara Federal Research Center of Russian Academy of Sciences, Institute of Ecology of the Volga River basin of RAS Togliatti Russia; 12 Perm State Humanitarian Pedagogical University, Perm, Russia Perm State Humanitarian Pedagogical University Perm Russia; 13 Central Chernozem Nature Reserve, Zapovednyi, Russia Central Chernozem Nature Reserve Zapovednyi Russia

**Keywords:** dataset, amphibians occurrences, Amphibia, data paper

## Abstract

**Background:**

Knowledge about the distribution of living organisms on Earth is very important for many areas of biological science and understanding of the surrounding world. However, much of the existing distributional data are scattered throughout a multitude of sources, such as taxonomic publications, checklists and natural history collections and often, bringing them together is difficult. A very successful attempt to solve this problem is the GBIF project, which allows a huge number of researchers to publish data in one place in a single standard. Our dataset represents a significant addition to the occurrences of amphibians in the Volga, Don riverine basins and adjacent territories.

The dataset contains up-to-date information on amphibian occurrences in the Volga river basin and adjacent territories, located for the most part on the Russian plain of European Russia. The dataset is based on our own studies that were conducted in the years 1996-2020. The dataset consists of 5,030 incident records, all linked to geographical coordinates. A total of 13 amphibian species belonging to nine genera and six families have been registered within the studied territory, although the distribution of amphibian species in this region of Russia has not yet been fully studied. This is especially relevant with the spread of cryptic species that can only be identified using molecular genetic research methods.

The main purpose of publishing a database is to make our data available in the global biodiversity system to a wide range of users. The data can be used by researchers, as well as helping the authorities to manage their territory more efficiently.

**New information:**

All occurrences are published in GBIF for the first time. Most of the data are stored in field diaries and we would like to make it available to everyone by adding it in the global biodiversity database (GBIF).

## Introduction

Amphibians are an important group of ectothermal animals that are particularly sensitive to global climate change and environmental conditions (Trochet et al. 2016, Kestemont 2019, Frishkoff et al. 2019, Préau et al. 2019, Chikhlyaev et al. 2020). Climate change will change the geographical ranges of species and have an effect on dispersal capacity (Duan et al. 2016, Johovic et al. 2020). Changes in climate conditions in recent years have also affected growth, reproduction, phenology, survival, dispersal, distribution, parasitic relationships, competitive interactions and food availability for individuals (Blaustein et al. 2002, Askenderov et al. 2018, Bosch et al. 2018, Lebedinskii et al. 2019, Vedernikov et al. 2020). The introduction of invasive species into reservoirs is also important for reproduction, as it influences the ability of amphibian populations to reproduce (Ruchin et al. 2019, Polo-Cavia et al. 2020). Climate change contributed to the expansion of the range of *Pelophylax ridibundus*, which is gradually spreading to the north and begins to occupy all habitats suitable for it. On the other hand, the abundance and range of *Salamandrella keyserlingii* in the study area is gradually decreasing due to climate warming. Tailed amphibians are also significantly influenced by the appearance of the fish *Perccottus glenii* in spawning reservoirs as this is capable of destroying their larvae (Reshetnikov and Karyagina 2015). Over the past 10 years, the population of *Bombina bombina* has decreased significantly. It can be assumed that this decrease is associated with weak floods, which do not fill the spawning reservoirs with water.

The Volga is the longest river in Europe and the 16th largest in the world. Its length is 3690 km. The area of the Volga basin is about 1.36 million km^2^, which is 33% of the territory of European Russia. There are different types of biomes in the Volga basin, such as taiga in the north and semi-desert in the south (Tockner et al. 2009). The Volga basin includes all or part of the territory of 37 regions of Russia. The Don is the third largest river in the European part of Russia with a length of 1870 km and area of 0.43 million km^2^. The main part of the Don basin is forest-steppe and steppe biomes, which are very heavily exposed to agricultural activities. This basin includes all or part of the territory of 14 regions of Russia.

This work can be considered the next stage in the study of distribution and abundance of amphibians for this territory after publication of "Materials for inventory of amphibians and reptiles of the Middle Volga" (Pestov 2002). In addition to new data on distribution and abundance of species, it has been established that species, previously considered *Pelobates fuscus*, consists of two cryptic species - *Pelobates fuscu*s and *Pelobates vespertinus*. The boundaries of their areas became clear, passing within the study area (Dufresnes et al. 2019b). The distribution of population systems of the Pelophylax esculentus group in the Volga basin also became clearer. A specific feature is the reduced occurrence (lower abundance) of *Pelophylax esculentus* here and relatively frequent occurrence of the REL-type population systems (Litvinchuk et al. 2020).

This study aims to describe a dataset consisting of up-to-date data on the occurrence of amphibians in the Volga and Don river basins (European Russia), which we have recently published in GBIF as the Darwin Core Archive (Ruchin 2020). This article was prepared as a "data paper" (Penev et al. 2017).

## Project description

### Title

Occurrence of the amphibians in the Volga and Don River basins (Russia): research in 1996-2020

### Study area description

Brief description of the Volga and Don River basins

## Sampling methods

### Quality control

Each observation contained fundamental information, such as location (coordinates), date, name of observer and name of identifier. A large part of the coordinates was determined directly on site with the help of a GPS device. In other cases, Google Maps (2020) were used. Species were identified according to Dunaev and Orlova (Dunaev and Orlova 2017). The main part of the species was determined at the site by external signs, as well as by acoustic methods, without killing the animal.

### Step description

The field names of the dataset were chosen according to Darwin Core (Wieczorek et al. 2012) and include the following: “occurrenceID”, “basisOfRecord”, scientificName”, “kingdom”, “phylum”, “class”, “order”, “family”, “coordinateUncertaintyInMeters”, “coordinatePrecision” “decimalLatitude”, “decimalLongitude”, “geodeticDatum”, “country”, “countryCode”, “individualCount”, “year”, “month”, “day”, “eventDate”, “recordedBy”, “identifiedBy”.

Geographical reference was made by fixing the coordinates of the meeting point of the amphibians using a GPS Navigator or using Google maps. The margin of error in the measurement of coordinates is 50 m. The accuracy of determining coordinates is up to the fourth digit. In all cases, the WGS-84 coordinate system is used.

## Geographic coverage

### Description

The dataset contains information about the occurrence of amphibians in 27 regions of Russia: the Chuvash Republic, the Republic of Mari-El, the Republic of Tatarstan, the Republic of Mordovia, the Republic of Kalmykia, the Republic of Udmurtia, the Republic of Komi, Perm Kray, Kaluga, Vladimir, Ryazan, Ivanovo, Tambov, Penza, Moscow, Voronezh, Kursk, Saratov, Samara, Astrakhan, Rostov, Lipetsk, Tula, Kirov, Orenburg, Ulyanovsk and Nizhny Novgorod regions.

**Figure 1.**
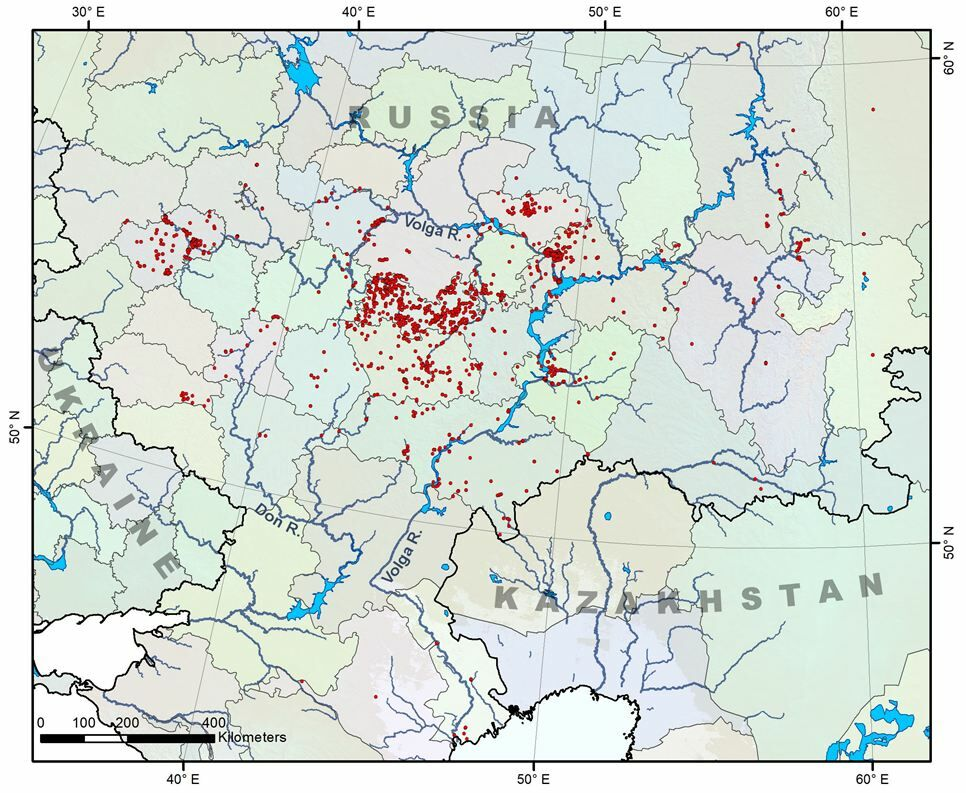
Collecting sites in the Volga and Don regions.

The study area is located within the Eastern European plain (Fig. 1). In the eEast, there is the Volga upland with maximum heights up to 350 m above sea level, in the West is the Central Russian upland (up to 300 m above sea level). Between them, there is the Oka-Don plain (up to 180 m above sea level). The territory is located in a temperate climate zone. The total duration of the period with an average daily air temperature below freezing is 140-150 days per year. The study area is divided into two different basins - the Black Sea basin (the Don river watershed) and the Caspian Sea basin (the Volga river watershed). All the rivers in the region are typically low-lying and belong to the Eastern European type. Its main characteristic is seasonal run-off. Spring floods occur in spring, water flow is minimal in summer and winter and river flow increases in autumn. Rivers have a mixed feed, which is made up of melting snow, precipitation and groundwater

The study area is crossed by the boundaries of the ranges of 11 species. In the east, the Kama basin passes the western part of the border area *Salamandrella keyserlingii*. The southern border of the distribution of many species (*Lissotriton vulgaris, Triturus cristatus, Bombina bombina, Bufo bufo, Rana arvalis, Rana temporaria*) coincides with the border of the middle and lower Volga ([Bibr B6427790][Bibr B6427798][Bibr B6454901]). The range of *Pelobates vespertinus* almost completely coincides with the boundaries of the study area, with the exception of the Bryansk and Kaluga regions in the west and Ural Mountains in the east (Bulakhova et al. 2020). In a small part of the eastern area, *Pelobates fuscus* is included in boundaries of the study area (Dufresnes et al. 2019b). The study area includes the western branch of the *Pelophylax lessonae* range, which also largely excludes the Lower Volga. The range of *Pelophylax esculentus* practically coincides with the range of *Pelophylax lessonae* ([Bibr B6427811][Bibr B6427827]).

### Coordinates

60°25'22.8" and 45°40'46.9" Latitude; 33°45'57.2" and 61°06'35.3" Longitude.

## Taxonomic coverage

### Description

All amphibian individuals were identified to species. The taxonomic diversity of the studied area is represented by 13 species belonging to six families from two orders. Given the scale of targeted studies of fauna, this is an almost exhaustive list of species that form natural self-reproducing populations.

### Taxa included

**Table:** 

Rank	Scientific Name	
species	*Salamandrella keyserlingii* Dybowski, 1870	
species	*Lissotriton vulgaris* (Linnaeus, 1758)	
species	*Triturus cristatus* (Laurenti, 1768)	
species	*Bombina bombina* (Linnaeus, 1761)	
species	*Pelobates fuscus* (Laurenti, 1768)	
species	*Pelobates vespertinus* (Pallas, 1771)	
species	*Bufo bufo* (Linnaeus, 1758)	
species	*Bufotes viridis* (Laurenti, 1768)	
species	*Pelophylax lessonae* (Camerano, 1882)	
species	*Pelophylax esculentus* (Linnaeus, 1758)	
species	*Pelophylax ridibundus* (Pallas, 1771)	
species	*Rana arvalis* (Nilsson, 1842)	
species	*Rana temporaria* Linnaeus, 1758	

## Usage licence

### Usage licence

Creative Commons Public Domain Waiver (CC-Zero)

## Data resources

### Data package title

Occurrence of the amphibians in the Volga and Don River basins (Russia): research in 1996-2020

### Resource link

https://www.gbif.org/dataset/88af45ca-a74e-4a6a-85e6-3d53683844b3

### Number of data sets

1

### Data set 1.

#### Data set name

Occurrence of the amphibians in the Volga and Don River basins (Russia): research in 1996-2020

#### Number of columns

22

#### Download URL

https://www.gbif.org/dataset/88af45ca-a74e-4a6a-85e6-3d53683844b3

#### 

**Data set 1.** 

Column label	Column description
occurrenceID	An identifier for the Occurrence (as opposed to a particular digital record of the occurrence)
basisOfRecord	Recommended best practice is to use the standard label of one of the Darwin Core classes
scientificName	The full scientific name, with authorship and date information, if known. When forming part of an Identification, this should be the name in the lowest level taxonomic rank that can be determined. This term should not contain identification qualifications, which should instead be supplied in the IdentificationQualifier term
kingdom	The full scientific name of the kingdom in which the taxon is classified
phylum	The full scientific name of the phylum or division in which the taxon is classified
class	The full scientific name of the class in which the taxon is classified
order	The full scientific name of the order in which the taxon is classified
family	The full scientific name of the family in which the taxon is classified
decimalLatitude	The geographic latitude (in decimal degrees, using the spatial reference system given in geodeticDatum) of the geographic centre of a Location. Positive values are north of the Equator, negative values are south of it. Legal values lie between -90 and 90, inclusive
decimalLongitude	The geographic longitude (in decimal degrees, using the spatial reference system given in geodeticDatum) of the geographic centre of a Location. Positive values are east of the Greenwich Meridian, negative values are west of it. Legal values lie between -180 and 180, inclusive
country	The name of the country or major administrative unit in which the Location occurs
countryCode	The standard code for the country in which the Location occurs
individualCount	The number of individuals represented present at the time of the Occurrence
year	The integer day of the month on which the Event occurred
geodeticDatum	The ellipsoid, geodetic datum or spatial reference system (SRS) upon which the geographic coordinates given in decimalLatitude and decimalLongitude are based.
coordinateUncertaintyInMeters	The horizontal distance (in metres) from the given decimalLatitude and decimalLongitude describing the smallest circle containing the whole of the Location. Leave the value empty if the uncertainty is unknown, cannot be estimated or is not applicable (because there are no coordinates). Zero is not a valid value for this term.
coordinatePrecision	A decimal representation of the precision of the coordinates given in the decimalLatitude and decimalLongitude.
month	The ordinal month in which the Event occurred
day	The integer day of the month on which the Event occurred
eventDate	The date-time or interval during which an Event occurred. For occurrences, this is the date-time when the event was recorded. Not suitable for a time in a geological context.
recordedBy	A person, group or organisation responsible for recording the original Occurrence
identifiedBy	A list (concatenated and separated) of names of people, groups or organisations who assigned the Taxon to the subject

## Additional information

This dataset contains up-to-date data on amphibian encounters in the Volga and Don river basins. The data set contains information for about 5,030 occurrences of 13 species (Ruchin 2020, Table 1).

## Figures and Tables

**Table 1.** Taxonomic composition of the dataset, number of observations (one species in one place) and number of specimens (registered individuals)

Taxa	Number of observations	Number of specimens
** Caudata **		
** Hynobiidae **		
*Salamandrella keyserlingii* Dybowski, 1870	3	3
** Salamandridae **		
*Lissotriton vulgaris* (Linnaeus, 1758)	371	1972
*Triturus cristatus* (Laurenti, 1768)	270	863
** Anura **		
** Bombinatoridae **		
*Bombina bombina* (Linnaeus, 1761)	231	1336
** Pelobatidae **		
*Pelobates fuscus* (Laurenti, 1768)	21	826
*Pelobates vespertinus* (Pallas, 1771)	384	1902
** Bufonidae **		
*Bufo bufo* (Linnaeus, 1758)	497	3954
*Bufotes viridis* (Laurenti, 1768)	295	1443
** Ranidae **		
*Pelophylax lessonae* (Camerano, 1882)	555	4574
*Pelophylax esculentus* (Linnaeus, 1758)	126	706
*Pelophylax ridibundus* (Pallas, 1771)	908	5868
*Rana arvalis* (Nilsson, 1842)	972	4688
*Rana temporaria* Linnaeus, 1758	397	11374
Total	5030	39509

## References

[B6372685] Askenderov A. D., Mazanaeva L. F., Mikhaylov R. A., Fayzulin A. I. (2018). Spawning water bodies and their role in conservation of rare amphibian species in the foothills of the Republic of Dagestan (Russia). Nature Conservation Research.

[B6372716] Blaustein A. R., Belden L. K., Olson D. H., Green D. M., Root T. L., Kiesecker J. M. (2002). Amphibian breeding and climate change. Conservation Biology.

[B6372727] Bosch J., Fernández-Beaskoetxea S., Garner T. W.J., Carrascal L. M. (2018). Long-term monitoring of an amphibian community after a climate change and infectious disease driven species extirpation. Global Change Biology.

[B6427207] Bulakhova Nina, Alfimov Arcady, Berman Daniil (2020). The eastern boundary of the geographic range of the Pallas’ spadefoot Pelobates
vespertinus (Anura, Amphibia) is limited by overwintering temperatures. Herpetozoa.

[B6372781] Chikhlyaev I. V., Ruchin A. B., Kirillov A. A. (2020). Ecological analysis of the helminth fauna in *Bufo
bufo* (Amphibia: Anura) from various habitats. Nature Conservation Research.

[B6372736] Duan R. Y., Kong X. Q., Huang M. Y., Varela S., Ji X. (2016). The potential effects of climate change on amphibian distribution, range fragmentation and turnover in China. PeerJ.

[B6427216] Dufresnes Christophe, Strachinis Ilias, Suriadna Nataliia, Mykytynets Galyna, Cogălniceanu Dan, Székely Paul, Vukov Tanja, Arntzen Jan W., Wielstra Ben, Lymberakis Petros, Geffen Eli, Gafny Sarig, Kumlutaş Yusuf, Ilgaz Çetin, Candan Kamil, Mizsei Edvárd, Szabolcs Márton, Kolenda Krzysztof, Smirnov Nazar, Géniez Philippe, Lukanov Simeon, Crochet Pierre-André, Dubey Sylvain, Perrin Nicolas, Litvinchuk Spartak N., Denoël Mathieu (2019). Phylogeography of a cryptic speciation continuum in Eurasian spadefoot toads (Pelobates). Molecular Ecology.

[B6372750] Dunaev E. A., Orlova V. F. (2017). Amphibians and reptiles of Russia: Determinant Atlas.

[B6427798] Faizulin A. I., Svinin A. O., Ruchin A. B., Skorinov D. V., Borkin L. J., Rosanov Y. M., Kuzovenko A. E., Litvichuk S. N. (2018). Distribution and contact zone of two forms of the green toad from the Bufotes
viridis complex (Anura, Amphibia), differing in genome size, in the Volga Region. Current Studies in Herpetology.

[B6427811] Fayzulin A. I., Zamaletdinov R. I., Litvinchuk S. N., Rosanov J. M., Borkin L. J., Ermakov O. A., Ruchin A. B., Lada G. A., Svinin A. O., Bashinsky I. V., Chikhlyaev I. V. (2018). Species composition and distributional peculiarities of green frogs (Pelophylax
esculentus complex) in Protected Areas of the Middle Volga Region (Russia). Nature Conservation Research.

[B6372902] Frishkoff L. O., Ke A., Martins I. S., Olimpi E. M., Karp D. S. (2019). Countryside biogeography: the controls of species distributions in human-dominated landscapes. Current Landscape Ecology Reports.

[B6427827] Ivanov A. Y., Ruchin A. B., Fayzulin A. I., Chikhlyaev I. V., Litvinchuk S. N., Kirillov A. A., Svinin A. O., Ermakov O. A. (2019). The first record of natural transfer of mitochondrial DNA from Pelophylax cf. bedriagae into P. lessonae (Amphibia, Anura). Nature Conservation Research.

[B6372951] Johovic I., Gama M., Banha F., Tricarico E., Anastácio P. M. (2020). A potential threat to amphibians in the European Natura 2000 network: forecasting the distribution of the American bullfrog *Lithobates
catesbeianus*. Biological Conservation. Biological Conservation.

[B6372961] Kestemont B. (2019). The bottom-up assessment of threatened species. Nature Conservation Research.

[B6427790] Kuzmin SL. (2012). Amphibians of the Former USSR.

[B6372970] Lebedinskii A. A., Noskova O. S., Dmitriev A. I. (2019). Post-fire recovery of terrestrial vertebrates in the Kerzhensky State Nature Biosphere Reserve (Central Volga Region, Russia). Nature Conservation Research.

[B6427247] Litvinchuk Spartak, Ivanov Alexander, Lukonina Svetlana, Ermakov Oleg (2020). A record of alien Pelophylax species and widespread mitochondrial DNA transfer in Kaliningradskaya Oblast’ (the Baltic coast, Russia). BioInvasions Records.

[B6373003] Penev L., Mietchen D., Chavan V., Hagedorn G., Smith V., Shotton D., Tuama E. O., Senderov V., Georgiev T., Stoev P., Groom Q., Remsen D., Edmunds S. (2017). Strategies and guidelines for scholarly publishing of biodiversity data. Research Ideas and Outcomes.

[B6427256] Pestov M. (2002). Материалы к кадастру амфибий и рептилий бассейна Средней Волги.

[B6373029] Polo-Cavia N., Boyero L., Martín-Beyer B., Navazo T., Bosch J. (2020). Effects of coexistence and predator experience on antipredatory responses of montane amphibian larvae towards native and introduced salmonids. Biological Invasions.

[B6373039] Préau C., Isselin-Nondedeu F., Sellier Y., Bertrand R., Grandjean F. (2019). Predicting suitable habitats of four range margin amphibians under climate and land-use changes in southwestern France. Regional Environmental Change.

[B6426520] Reshetnikov Andrey N., Karyagina Anna S. (2015). Further evidence of naturalisation of the invasive fish Perccottus
glenii Dybowski, 1877 (Perciformes: Odontobutidae) in Germany and necessity of urgent management response. ACTA ZOOLOGICA BULGARICA.

[B6388091] Ruchin A. (2020). Occurrence of the amphibians in the Volga and Don River basins (Russia): research in 1996-2020. Occurrence dataset. https://www.gbif.org/dataset/88af45ca-a74e-4a6a-85e6-3d53683844b3.

[B6373049] Ruchin A. B., Osipov V. V., Fayzulin A. I., Bakin O. V., Tselishcheva L. G., Bayanov N. G. (2019). Chinese sleeper (*Perccottus
glenii* Dybowski, 1877) (Pisces, Odontobutidae) in the reserves and National Parks of the middle and lower Volga (Russia): mini-review. AACL Bioflux.

[B6373060] Tockner K., Robinson C. T., Uehlinger U. (2009). Rivers of Europe.

[B6372672] Trochet A., Dechartre J., Chevalier H. L., Baillat B., Calvez O., Blanchet S., Ribéron A. (2016). Effects of habitat and fragmented-landscape parameters on amphibian distribution at a large spatial scale. Herpetological Journal.

[B6373068] Vedernikov A. A., Svinin A. O., Ermakov O. A., Chelyadnikova Y. A., Musatov G. A., Drobot G. P. (2020). Granulomatous inflammations in the intestine of *Pelophylax
ridibundus* (Anura: Ranidae) caused by *Brandesia
turgida* (Plathelminthes: Digenea). Nature Conservation Research.

[B6454901] Zaks M. M., Simonov E. P., Ermakov O. A. (2011). Distribution of amphibians in Penza region. Izv. Penz. gos. pedagog. univ. im.i V.G. Belinskogo.

